# Salt-Mediated Au-Cu Nanofoam and Au-Cu-Pd Porous Macrobeam Synthesis

**DOI:** 10.3390/molecules23071701

**Published:** 2018-07-12

**Authors:** Fred J. Burpo, Enoch A. Nagelli, Lauren A. Morris, Kamil Woronowicz, Alexander N. Mitropoulos

**Affiliations:** 1 Department of Chemistry and Life Science, United States Military Academy, West Point, NY 10996, USA; enoch.nagelli@usma.edu (E.A.N.); kamil.woronowicz@usma.edu (K.W.); alex.mitropoulos@usma.edu (A.N.M.); 2 Armament Research, Development and Engineering Center, U.S. Army RDECOM-ARDEC, Picatinny Arsenal, NJ 07806, USA; lauren.a.morris17.civ@mail.mil; 3 Department of Mathematical Sciences, United States Military Academy, West Point, NY 10996, USA

**Keywords:** nanomaterials, porous, gold, copper, palladium

## Abstract

Multi-metallic and alloy nanomaterials enable a broad range of catalytic applications with high surface area and tuning reaction specificity through the variation of metal composition. The ability to synthesize these materials as three-dimensional nanostructures enables control of surface area, pore size and mass transfer properties, electronic conductivity, and ultimately device integration. Au-Cu nanomaterials offer tunable optical and catalytic properties at reduced material cost. The synthesis methods for Au-Cu nanostructures, especially three-dimensional materials, has been limited. Here, we present Au-Cu nanofoams and Au-Cu-Pd macrobeams synthesized from salt precursors. Salt precursors formed from the precipitation of square planar ions resulted in short- and long-range ordered crystals that, when reduced in solution, form nanofoams or macrobeams that can be dried or pressed into freestanding monoliths or films. Metal composition was determined with X-ray diffraction and energy dispersive X-ray spectroscopy. Nitrogen gas adsorption indicated an Au-Cu nanofoam specific surface area of 19.4 m^2^/g. Specific capacitance determined with electrochemical impedance spectroscopy was 46.0 F/g and 52.5 F/g for Au-Cu nanofoams and Au-Cu-Pd macrobeams, respectively. The use of salt precursors is envisioned as a synthesis route to numerous metal and multi-metallic nanostructures for catalytic, energy storage, and sensing applications.

## 1. Introduction

High surface area and volume-to-mass ratios and the ability to tune reaction specificity through the variation of metal composition facilitate the use of bi-metallic and alloy nanomaterials for a broad range of catalytic applications [1,2,3,4,5]. Synthesizing these materials as three-dimensional nanostructures enables control of surface area, pore size and mass transfer properties, and electronic conductivity. Further, monolithic, freestanding three-dimensional nanostructures allow for device integration without the need for added support and binding materials and mitigate nanoparticle agglomeration with prolonged use [6,7]. Freestanding three dimensional noble metal and multi-metallic nanostructures are promising materials for electro-optical sensing, catalysis, and biomedical applications [8].

Gold nanoparticles have been used for a range of organic reaction catalysis [9,10,11,12], CO oxidation [13,14,15,16], and electrocatalytic reduction of CO_2_ [17]. Copper nanoparticles have been examined for their use in the electrocatalytic reduction of CO_2_ [18,19,20,21,22], carbon-carbon and carbon-heteroatom bond formation [23,24,25], and dehydrogenation reactions [26]. The introduction of copper to gold nanoparticles has the potential to tune plasmonic and catalytic behavior, as well as to reduce the material cost by lowering the relative amount of gold [27]. Gold-copper nanomaterials, in particular, have been used for tunable plasmonics [28,29,30], methanol oxidation [31], CO oxidation [32], electrocatalytic reduction of CO_2_ [33], and oxygen reduction reaction (ORR) [34]. The number of reports and synthesis methods for Au-Cu bi-metallic and alloy structures has been limited. Au-Cu bi-metallic, alloy, and core shell nanoparticles have been prepared via chemical reduction with surfactants [35], photochemical formation of Au-Cu nanoparticles [36], pulsed laser targeting and stirring [37], and sequential electrochemical deposition for core shell nanocubes [29]. Au-Cu nanoparticle assembly through sol-gel preparation has been demonstrated to achieve three dimensional assemblies [38]. Our group’s recent reports on direct reduction synthesis of noble metal aerogels [39] and salt-templated platinum macrobeams [40] suggest a possible route to achieving bi- and tri-metallic three-dimensional Au-Cu and Au-Cu-Pd nanostructures. The use of these synthesis methods in conjunction with biotemplating has also demonstrated the versatility of these approaches to yield porous noble metal structures [41,42].

Here, we present a synthesis method for Au-Cu nanofoams and Au-Cu-Pd macrobeams using salt crystal precursors. The combination of oppositely charged square planar ion complexes yields short- and long-range ordered salt needles. When the salt precursors are reduced in solution, Au-Cu salts yield nanofoams, while Au-Cu-Pd salts yield macrobeams, both of which may be pressed into free standing films. Metal composition was determined with X-ray diffractometry and energy dispersive X-ray spectroscopy. Electrochemical impedance spectroscopy was used to determine specific capacitances and corresponding electrochemically active surface areas for Au-Cu nanofoams and Au-Cu-Pd macrobeams. Characteristic redox peaks determined by cyclic voltammetry indicated these materials’ potential for catalysis. The use of insoluble salt precursors is envisioned as a route to synthesize a broad range of metal and multi-metallic nanostructures for numerous catalytic and sensing applications.

## 2. Materials and Methods

### 2.1. Au-Cu Nanofoam and Au-Cu-Pd Macrobeam Synthesis

Salt precursors for Au-Cu nanofoams were formed by combining equal volumes of 100 mM HAuCl_4_•3H_2_O (Sigma Aldrich, St. Louis, MO, USA) and Cu(NH_3_)_4_SO_4_•H_2_O (Sigma Aldrich, St. Louis, MO, USA) for total volumes between 1–10 mL. Salt precursors for Au-Cu-Pd macrobeams were formed by combining 100 mM Cu(NH_3_)_4_SO_4_•H_2_O and Pd(NH_3_)_4_Cl_2_•H_2_O (Sigma Aldrich, St. Louis, MO, USA) at 1:1 (*v*/*v*) to form a copper-palladium cation solution. The copper-palladium cation solution was then added to 100 mM HAuCl_4_•3H_2_O at 1:1 (*v*/*v*) to form precursor salt needles. Au-Cu and Au-Cu-Pd salt precursors were each added to 100 mM dimethylamine borane (DMAB) reducing agent at a 4:50 (*v*/*v*) salt solution to reducing agent in 50 mL reaction volumes. Chemical reduction proceeded for 18 h. The resulting metal structures were rinsed in deionized water for 12–24 h and dried at ambient temperature. Au-Cu nanofoams were either dried in a microfuge tube to form a monolith, or pressed by hand in between glass slides at approximately 200 kPa to form a film. Au-Cu-Pd macrobeams were also pressed by hand between glass slides to form freestanding films.

### 2.2. Polarized Optical Microscopy

Salt precursors were imaged by polarized optical microscopy with an AmScope PZ300JC polarized light microscope (AmScope, Irvine, CA, USA). ImageJ was used to measure salt needle lengths [43].

### 2.3. Scanning Electron Microscopy

Nanofoam and macrobeam structure was imaged with a FEI Helios 600 scanning electron microscope (SEM) (Thermo Fisher Scientific, Hillsboro, OR, USA). Energy dispersive X-ray spectroscopy (EDS) was performed with a Bruker Quantax 70 EDX as part of a Hitachi TM-3000 SEM (Hitachi, Tarrytown, NJ, USA). Samples were imaged after drying at ambient conditions and placement on carbon tape.

### 2.4. X-ray Diffractometry

X-ray diffractometry (XRD) spectra were collected with a PANalytical Emperean diffractometer (PANalytical, Almelo, The Netherlands). Diffraction angles (2θ) from 5° to 90°, with a 2θ step size of 0.0130°, and 20 s per step were used at 45 kV and 40 mA with Cu K_α_ radiation (1.54060 Å). High Score Plus software (PANalytical) was used for XRD spectra analysis. Crystallite size (D) was determined with the Debye-Scherrer formula,

D = Kλ/(Bcosθ)(1)

with the shape factor (K), full width at half maxima (B), radiation wave length (λ), and Bragg angle (θ). A shape factor of K = 0.9 was used for crystallite size determination. After drying at ambient conditions, samples were placed on a glass slide and gently pressed to form a thin film for analysis.

### 2.5. X-ray Fluorescence

X-ray fluorescence (XRF) was performed with a Rigaku NEX CG (Rigaku, The Woodlands, TX, USA). Au-Cu and Au-Cu-Pd salt samples were prepared as indicated in Section 2.1 and then centrifuged with a Thermo Scientific Heraeus Pico 21 (Thermo Fisher Scientific, Waltham, MA, USA) at 21.1 xG. The supernatants were removed, and the salts were resuspended in deionized water. Centrifugation and resuspension in fresh deionized water was repeated for three cycles.

### 2.6. UV-VIS

Au-Cu nanofoams and Au-Cu-Pd macrobeams were sonicated using Fisherbrand Model 50 Sonic Dismembrator (Thermo Fisher Scientific, Waltham, MA, USA) on a 100% setting for 10 s prior to UV-VIS absorbance measurements. Absorbance and fluorescence was collected on a Take 3, BioTek (BioTek, Winooski, VT, USA) multi-volume plate with 2 µL sample volumes on a Synergy 4 (BioTek) microplate reader.

### 2.7. BET Analysis

Au-Cu nanofoams were analyzed after drying at ambient conditions. Adsorption–desorption measurements were performed according to IUPAC standards [44] using a Quantachrome NOVA 4000e (Quantachrome Instruments, Boynton Beach, FL, USA) surface area and pore size analyzer with nitrogen (−196 °C) as the test gas. All samples were vacuum degassed at room temperature for 12 h prior to measurement. Brunauer–Emmett–Teller (BET) analysis [45] was used to determine the specific surface area from gas adsorption. Pore-size distributions for each sample were calculated using the Barrett–Joyner–Halenda (BJH) model [46] applied to volumetric desorption isotherms. All calculations were performed using Quantachrome’s NovaWin software (Version 11.04, Quantachrome Instruments, Boynton Beach, FL, USA).

### 2.8. Electrochemical Characterization

Electrochemical impedance spectroscopy (EIS) and cyclic voltammetry (CV) were performed with a Bio-Logic VMP-3 potentiostat (Bio-Logic, Knoxville, TN, USA). An Ag/AgCl reference electrode, a 1 mm platinum wire counter-electrode, and a lacquer coated 1 mm platinum wire with exposed tip in contact with samples were used in a three electrode cell with 0.5 M H_2_SO_4_ electrolyte. EIS was performed at open circuit voltage with a frequency range of 1 MHz to 1 mHz with a 10 mV sine wave. CV was performed in a voltage range of −0.2 to 1.2 V (vs. Ag/AgCl) with scan rates of 0.5, 1, 5, 10, 25, 50, 75, and 100 mV/s.

## 3. Results and Discussion

### 3.1. Nanofoam and Porous Macrobeam Synthesis

**Figure 1:**
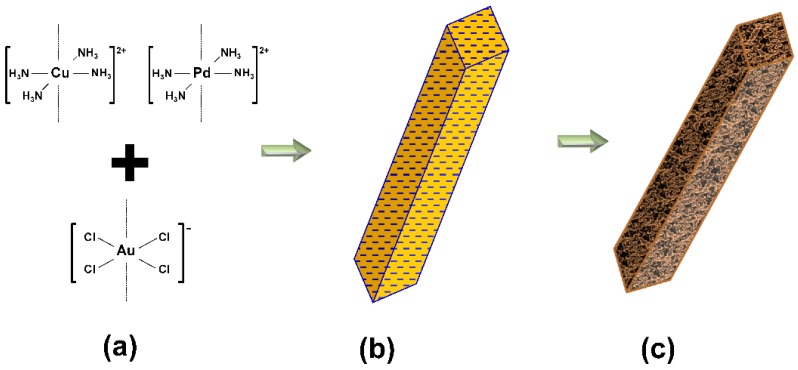
Nanofoam and macrobeam synthesis scheme. (**a**) Addition of [AuCl_4_]^−^ with [Cu(NH_3_)_4_]^2+^ and [Pd(NH_3_)_4_]^2+^; (**b**) Linear stacking and precipitation of square planar complex ions forming salt crystal precursors; (**c**) Reduction of Au-Cu-Pd salt precursor to a porous macrobeam.

Figure 1 depicts the synthesis scheme to form Au-Cu and Au-Cu-Pd salt precursors, and with subsequent chemical reduction, nanofoams and macrobeams, respectively. The combination of oppositely charged square planar ion complexes (Figure 1a) rapidly forms insoluble salt needles with short- and long-range order (Figure 1b). Mixing stoichiometrically equivalent amounts of [AuCl_4_]^−^ and [Cu(NH_3_)_4_]^2+^ ions forms salts with short-range, high aspect ratio salt crystals. Long salt needles form when molar equivalent quantities of [Pd(NH_3_)_4_]^2+^ and [Cu(NH_3_)_4_]^2+^ cations are added to negatively charged [AuCl_4_]^−^ anions, as represented in Figure 1b. Chemical reduction of these salt precursors results in nanofoams for Au-Cu salts, and porous macrobeams for Au-Cu-Pd salts schematized in Figure 1c.

**Figure 2:**
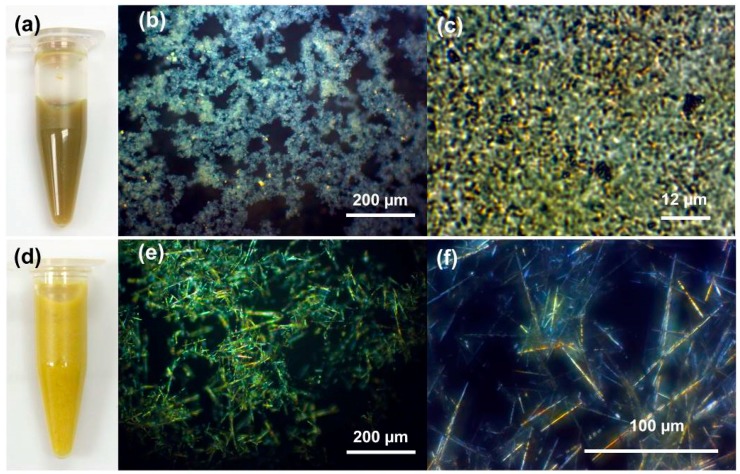
(**a**) Photograph of Au-Cu salt precursors in solution; (**b**,**c**) Polarized optical microscope (POM) images of Au-Cu salt precursors; (**d**) Photograph of Au-Cu-Pd salt precursors in solution; (**e**,**f**) POM images of Au-Cu-Pd salt precursors.

Figure 2a,d show photo images of the Au-Cu and Au-Cu-Pd salt precursors, respectively. The Au-Cu insoluble salts present a yellow-brown color in solution, while the Au-Cu-Pd salts appear yellow. Polarized optical microscopy (POM) images of the Au-Cu salts are shown in Figure 2b,c. These salts appear randomly oriented over a long-range of 100’s of micrometers (Figure 2b), yet at higher magnification present short-range order high aspect ratio salt crystals from 1–2 µm long (Figure 2c). To explore the possibility of tri-metallic nanostructure synthesis, the introduction of square planar palladium cations to copper cations resulted in salt needles 10 s to 100 s of micrometers long when added to square planar gold anions (Figure 2e,f). Both Au-Cu and Au-Cu-Pd salts present birefringence under polarized light indicating anisotropic crystalline lattice structure.

### 3.2. Scanning Electron Microscopy

**Figure 3:**
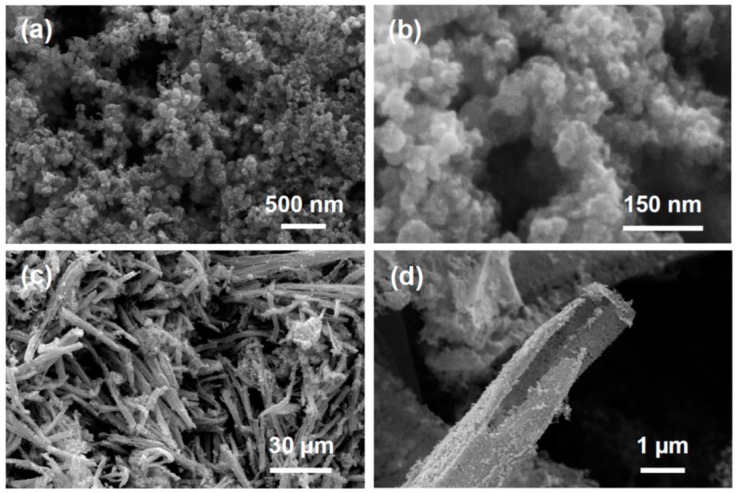
Scanning electron microscope images of (**a**,**b**) Au-Cu bi-metallic porous nanofoam; (**c**,**d**) Porous Au-Cu-Pd macrobeams.

Figure 3 shows scanning electron micrographs of metalized Au-Cu and Au-Cu-Pd salt precursors after chemical reduction with 100 mM DMAB reducing agent. Figure 3a,b shows the resulting nanofoam structure from the reduction of Au-Cu salts (nanofoam photos are shown in Figure S1). The nanofoam is composed of interconnected nanoparticles ranging in diameter from 5–50 nm with an average diameter of 13.4 ± 8.4 nm. Pore structure ranges from 1 to 10’s of nanometers, presenting a mix of mesopores (between 2–50 nm) and macropores (>50 nm). Reduction of the Au-Cu-Pd precursor salts results in high aspect ratio macrobeams with porous sidewalls as seen in Figure 3c,d. The macrobeams are composed of fused nanoparticles ranging in diameter from 10–35 nm with an average diameter of 21.6 ± 4.4 nm (determined from Figure S3). These fused nanoparticles form the flat porous sidewalls of the macrobeams as seen Figure 3d. These macrobeams present a similar structure to the previous report for salt-templated platinum macrobeams [40]. The porous macrobeams seen in Figure 3c,d are believed to result from the reduction-dissolution mechanism previously described [40]. In this case, the reduction of the gold chloride anion results in four chloride ions entering in solution as seen in Equation (2).

[AuCl_4_]^−^ (aq) + 3 e^−^ ↔ Au(s) + 4 Cl^−^ (aq) (2)

Consequently, in order to maintain electroneutrality in the solution, the cations within the salt precursor are believed to dissolve out of the salt crystal and into solution [40]. For the Au-Cu salt precursor, two [Cu(NH_3_)_4_]^2+^ ions would dissolve to electrostatically balance the four chloride ions from the reduction of one [AuCl_4_]^−^ ion. The copper ions dissolved into solution are then available for subsequent reduction and fusing into the macrobeam structure. In this manner, the reducing agent in the solution may penetrate deeper into the salt precursor as it is both reducing and dissolving. Surface free energy minimization and Ostwald ripening is believed to contribute to the nanoparticle fusion observed in Figure 3 [47].

Elemental composition analysis determined with energy dispersive X-ray spectroscopy (EDS) indicated that Au-Cu nanofoams had an Au:Cu atomic ratio of 1:0.98, closely matching the ion stoichiometry of the precursor salt solutions. Given the differing charges on the [AuCl_4_]^−^ and [Cu(NH_3_)_4_]^2+^ ions precipitating as the insoluble salt precursor, a 2:1 gold to copper ratio would be expected for the salts. EDS spectra of salts rinsed in deionized water indicated an Au:Cu ratio of 10:1 (XRF indicated a 20:1 ratio). This suggests that copper ions in solution not precipitated into the salt precursor are also reduced into nanoparticles and fuse with the nanofoam structure. For the metal macrobeams, EDS spectra indicated an Au:Cu:Pd ratio of 3.7:1:1.3 suggesting that the elemental composition approximately matches the initial solution ion stoichiometry of 2:1:1. However, EDS of the rinsed Au-Cu-Pd salts indicated an elemental ratio of 1.6:0.04:1 for Au:Cu:Pd (XRF ratio was 3.5:0.06:1). The EDS and XRF spectra indicate minimal inclusion of copper ions into the precursor insoluble salts, suggesting that copper in the reduced metal macrobeams originates from bulk solution.

### 3.3. X-ray Diffractometry

**Figure 4:**
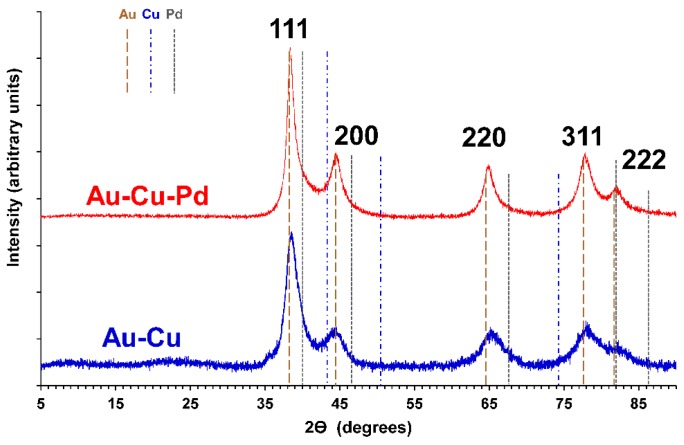
X-ray diffraction spectra of Au-Cu nanofoams and Au-Cu-Pd macrobeams. Peaks were indexed to JCPDS reference number 01-071-4615 for Au (brown dashed lines), 03-065-9743 for Cu (blue dashed lines), and 01-088-2335 for Pd (gray dashed lines). Dashed line heights are equal for each Miller index.

The X-ray diffraction spectra for Au-Cu nanofoams and Au-Cu-Pd macrobeams are shown in Figure 4 for a 2θ range of 5° to 90°. The peaks for both Au-Cu and Au-Cu-Pd most closely align with the Joint Committee on Powder Diffraction Standards (JCPDS) reference number 01-071-4615 for Au. The JCPDS Au peaks are identified with brown dashed lines for Miller indices 111, 200, 220, 311, and 222. While the spectra exhibit a strong Au character, the indexed Au peaks do not perfectly align. Cu peaks for JCPDS reference number 03-065-9743 are shown with blue dashed lines, and Pd peaks for reference number 01-088-2335 are shown with gray dashed lines. Indexed peaks for each Miller index are the same height for all three metals. For the Au-Cu nanofoam, the Cu 111 peak at 43.4° lies between the 111 and 200 peaks for Au at indexed positions of 38.2° and 44.5°, respectively. The convolution of Au and Cu 111 peaks suggest the reason for the measured 111 peak positioned more positive than the indexed Au peak. Similarly, the copper 220 indexed peak at 74.3° contributes to the positive shift in the measured 220 peak at 65.5° relative to the indexed Au 220 peak at 64.7°. The contribution of both Cu and Pd for the Au-Cu-Pd macrobeams shift the measured peaks from the indexed Au peaks in a similar manner. All three metals have a Fm-3m space group and cubic crystal system. Crystallite sizes for Au-Cu and Au-Cu-Pd determined from 111 XRD peaks with the Debeye-Sherrer formula were 8.5 nm and 15.6 nm, respectively. These crystallite sizes generally correlate to the feature sizes determined from SEM image analysis with average particle sizes determined from image analysis approximately 5 nm larger than crystallite size in each case.

### 3.4. UV-VIS

**Figure 5:**
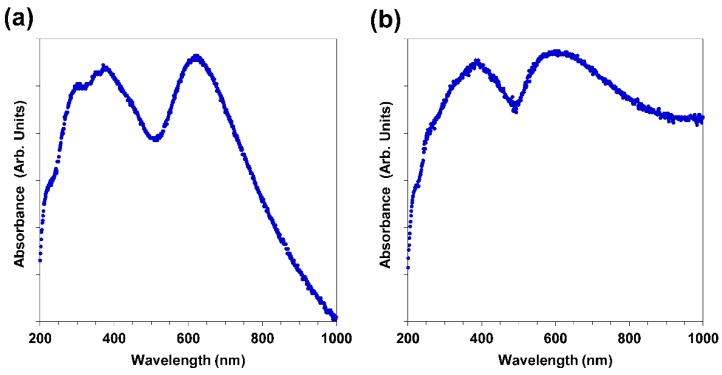
UV-VIS spectra of (**a**) Au-Cu nanofoams; and (**b**) Au-Cu-Pd macrobeams.

To determine the UV-VIS absorbance spectra for the Au-Cu and Au-Cu-Pd samples shown in Figure 5, the nanofoams and macrobeams were briefly sonicated to achieve a nanoparticle suspension in water. The nanofoam and macrobeam particle mass resulted in sedimentation and erratic spectra without sonication; however, the authors acknowledge the potential alteration of nanoparticle morphology and plasmonic response change that may have resulted from sonication. For Au-Cu nanofoams (Figure 5a), peaks were observed at 300 nm, 378 nm, and 620 nm. The broad Au peak centered at approximately 620 nm and ranging from 520 to 700 nm suggests Au nanoparticles as small as 10 nm with large nanoparticles and/or agglomerates with diameters in excess of 100 nm [48]. Au-Cu alloy nanoclusters have also been previously observed to exhibit a surface plasmon resonance band at 530 nm [49]. The broad superimposed peaks centered at 300 nm and 378 nm are attributed to a combination of copper [50] and possible copper oxide nanoparticles [51].

The UV-VIS spectrum for Au-Cu-Pd macrobeams in Figure 5b exhibits broad absorption peaks at 388 nm and 600 nm, and as in the case of Au-Cu nanofoams are attributed to Au and Cu nanoparticles in combination with the presence of Pd. The absorbance spectra extend into the near IR region, where surface plasmon peaks have been previously reported for anisotropic gold nanoparticles in the near IR range [52,53]. Anisotropic platinum fibrils were previously observed on salt-templated platinum macrobeams, and the fused nanoparticles comprising the Au-Cu-Pd macrobeam sidewalls present a linear “beads-on-a-string” appearance (Figure S3) and may contribute to this near IR absorbance.

### 3.5. Nitrogen Gas Adsorption

**Figure 6:**
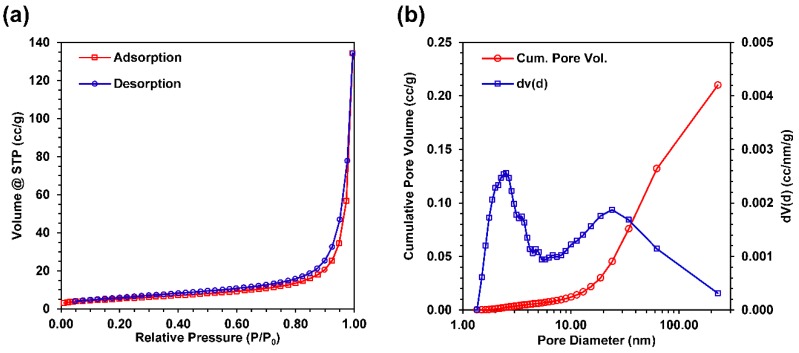
Gas adsorption data for Au-Cu nanofoam sample. (**a**) Complete nitrogen adsorption–desorption isotherms; (**b**) Pore size distribution with cumulative pore volume calculated by BJH analysis.

The BET-specific surface area of the Au-Cu nanofoam was 19.432 m^2^/g. The physisorption data shown in Figure 6a illustrates type IV adsorption-desorption isotherms in accordance with the IUPAC classification standards, revealing both mesoporous and macroporous structures in the nanofoam. The nitrogen adsorption quantity rises sharply at high relative pressures and no limiting adsorption is observed, consistent with the presence of both mesopores (2–50 nm in diameter) and macropores (>50 nm in diameter). At high relative pressure, P/P_0_ = 0.995, the maximum volume adsorbed for the Au-Cu nanofoam was 134.3 cc/g. H3 type hysteresis in the adsorption-desorption isotherms is characteristic of capillary condensation in the mesopores. The hysteresis extends to very low relative pressures (P/P_0_ as low as 0.05), indicating a large number of mesopores (<50 nm) present. BJH analysis (Figure 6b) of the desorption curve shows that that the largest fraction of pores are mesopores less than 50 nm in diameter, with the highest frequency of pores centered around 2 nm. This result is consistent with the hysteresis observed in the adsorption-desorption isotherms. BJH analysis also reveals that the total cumulative pore volume for the Au-Cu nanofoam was 0.210 cc/g and that the cumulative pore volume of pores less than 50 nm was approximately 0.110 cc/g. Therefore, approximately 52% of the total pore volume is attributed to mesopores. Given the relative volumes of a mesopore as compared to a macropore, it is evident that there is a much larger frequency of mesopores than macropores in the Au-Cu sample. The gas adsorption and pore size analysis results seem consistent with the SEM images of the samples (Figure 3a,b). The large macropores observed at low magnification gives the samples a relatively low overall BET specific surface area considering the high number of mesopores present. However, at high magnification the nanofoam surface is observed to be a connected network of particles well under 100 nm in diameter, which is highly mesoporous. Given the large macroporous content for the Au-Cu-Pd macrobeams evident in Figure 3 SEM images, nitrogen gas physisorption was not performed for those samples. Electrochemically active surface area (ECSA) for Au-Cu-Pd macrobeams was determined using electrochemical impedance spectroscopy (EIS) in the following section.

### 3.6. Electrochemical Characterization

**Figure 7:**
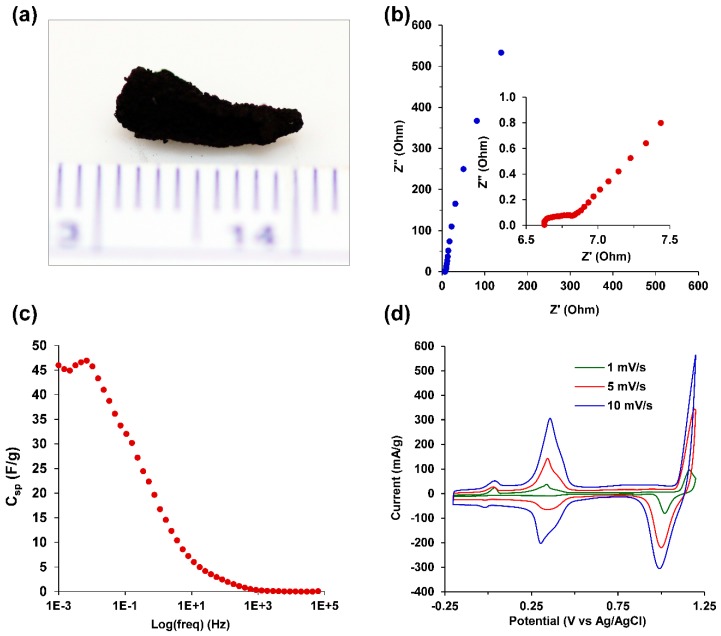
Electrochemical characterization of Au-Cu nanofoams. (**a**) Au-Cu nanofoam dried at ambient temperature; (**b**) Potentiostatic electrochemical impedance spectroscopy (PEIS) in 0.5 M H_2_SO_4_ at frequency range of 65 kHz to 1 mHz; (inset) high frequency EIS from 65 kHz to 2.5 Hz; (**c**) Specific capacitance, C_sp_ (F/g), versus log(frequency) (Hz); (**d**) CV in 0.5 M H_2_SO_4_ for scan rates of 1, 5, and 10 mV/s.

**Figure 8:**
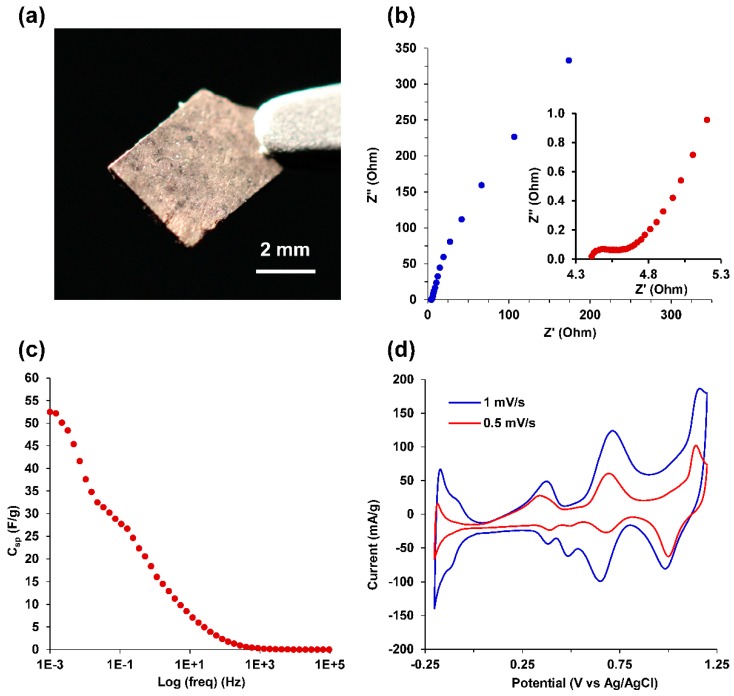
Electrochemical characterization of Au-Cu-Pd macrobeams. (**a**) Au-Cu-Pd porous film; (**b**) Potentiostatic electrochemical impedance spectroscopy (PEIS) in 0.5 M H_2_SO_4_ at frequency range of 65 kHz to 1 mHz; (inset) high frequency EIS from 65 kHz to 1 Hz; (**c**) Specific capacitance, C_sp_ (F/g), versus log(frequency) (Hz); (**d**) CV in 0.5 M H_2_SO_4_ for 0.5 mV/s and 1 mV/s scan rates.

After chemical reduction and rinsing, the bimetallic Au-Cu nanofoams were prepared by drying at ambient temperature resulting in a compacted monolith (Figure 7a) or by pressing at approximately 200 kPa between glass slides into freestanding films for electrochemical characterization. Au-Cu-Pd macrobeams were pressed in a similar manner for electrochemical characterization, as shown in Figure 8a. Electrochemical impedance spectroscopy (EIS) was performed in 0.5 M H_2_SO_4_ over a frequency range of 100 kHz to 1 mHz, with the resulting Nyquist plots shown in Figure 7b and Figure 8b for Au-Cu and Au-Cu-Pd films, respectively. Both the Au-Cu nanofoam and Au-Cu-Pd macrobeam films exhibit characteristic high surface area capacitive behavior throughout the whole frequency range. Figure 7b and Figure 8b insets depict the high frequency response associated with capacitive transmission line model behavior of porous materials [54,55]. EIS transmission line model fitting is shown in Figure S5.

Specific capacitances (C_sp_) were determined from the low frequency region of the EIS spectra in Figure 7b and Figure 8b using the relation,

C_sp_ = 1/(2πZ″*f*m) (3)

where *f* is the frequency, Z″ is the imaginary component of impedance, and m is the sample mass. Specific capacitance as a function of frequency for the Au-Cu nanofoam is plotted in Figure 7c and in Figure 8c for the Au-Cu-Pd macrobeams. At 1 mHz, C_sp_ for the Au-Cu nanofoam was 46.0 F/g and 52.5 F/g for the Au-Cu-Pd macrobeam film. The equivalent specific electrochemically active surface area (ECSA), using a nominal 30 µF/cm^2^ for planar metal capacitance, is 153 m^2^/g and 175 m^2^/g for Au-Cu and Au-Cu-Pd, respectively [56]. Using the nanofoam and macrobeam mass ratios from EDS analysis, the idealized nanoparticle diameters are approximately 2.8 nm and 2.2 nm, respectively. These idealized diameters suggest feature sizes and particle agglomerate porosities smaller than those resolved with SEM image analysis, but consistent with the high frequency of approximately 2 nm pore sizes seen with nitrogen gas physisorption in Figure 6b.

Cyclic voltammetry (CV) of the Au-Cu nanofoam and the Au-Cu-Pd macrobeam films was conducted in 0.5 M H_2_SO_4_ and shown for scan rates of 1, 5, and 10 mV/s in Figure 7d, and 0.5 and 1 mV/s in Figure 8d, respectively. Given that the films may be pressed to any arbitrary thickness, current values were normalized to mass with values given as mA/g. The CV scan for the Au-Cu nanofoam clearly indicates the presence of a bimetallic nanostructures with redox peaks centered at +1.11 V, +0.34 V, and –0.01 V (vs. Ag/AgCl) associated with Au/Au^3+^, Cu/Cu^+^ and Cu^+^/Cu^2+^ redox couples, respectively. The CV shows a pronounced oxidation peak at +1.16 V (vs. Ag/AgCl) from the adsorption of oxygen species onto Au coupled with a sequential reduction peak of the Au-oxide at +1.02 V (vs. Ag/AgCl) [57,58]. The oxidation peaks at +0.03 V and +0.34 V (vs. Ag/AgCl) relate to the oxidation of Cu^+^ to Cu^2+^, and Cu(s) to Cu^+^, respectively [59,60]. The distinct reduction peak at +0.34 V (vs. Ag/AgCl) is from reduction of Cu^+^ to Cu(s), and reduction peak present at –0.01 V (vs. Ag/AgCl) is due to the reduction of Cu^2+^ to Cu^+^. The difference in peak currents at those specific redox potentials associated with the oxidation-reduction of Cu are related to the relative amounts of specific copper oxide that is present in the nanofoam [59,60].

The CV scans in Figure 8d for Au-Cu-Pd macrobeams indicate characteristic hydrogen adsorption and desorption peaks associated with the presence of palladium between –0.2 and 0 V (vs. Ag/AgCl). The oxidation peaks observed at +0.38 V, +0.70 V, and +1.1 V (vs. Ag/AgCl) are attributed to the oxidation of Cu/Cu^+^, Pd/Pd^2+^, and Au/Au^3+^ metal phases, respectively. The reduction peaks at 0.99 V, 0.65 V, and 0.38 V (vs. Ag/AgCl) are attributed to the reduction of Au^3+^/Au, Pd^2+^/Pd, and Cu^+^/Cu. The reduction peak at 0.49 V (vs. Ag/AgCl) is attributed to the reduction of oxygen in acidic solution to H_2_O_2_.

## 4. Conclusions

Here we have demonstrated the use of insoluble salt precursors to mediate the synthesis of porous Au-Cu nanofoams and Au-Cu-Pd macrobeams. Nanofoams exhibit high surface area and mesoporous structure from nitrogen adsorption measurements. Au-Cu nanofoams and Au-Cu-Pd macrobeams provide specific capacitances of 40.6 F/g and 52.5 F/g and specific electrochemically active surface areas of 153 m^2^/g and 175 m^2^/g, respectively, and when pressed into freestanding films will ultimately enable practical device integration. The use of salt precursors to synthesize bi-metallic Au-Cu and tri-metallic Au-Cu-Pd porous materials in a single solution-based reduction step further demonstrates the applicability of this method for metal and multi-metallic porous nanostructures. With the ability to rapidly achieve high surface area materials, we envision this method will be useful to further develop catalytic, energy storage and conversion, and sensor materials.

## Supplementary Materials

The following are available online, Figure S1: Photo images of nanofoams prepared from Au-Cu salt precursors, Figure S2: EDS spectra of Au-Cu nanofoams and Au-Cu-Pd macrobeams, Figure S3: SEM image of Au-Cu-Pd macrobeam porous sidewall, Figure S4: UV-VIS spectra for Au-Cu and Au-Cu-Pd salts. Figure S5: EIS transmission line model fitting.
