# Supplementary material for: Salt-Mediated Au-Cu Nanofoam and Au-Cu-Pd Porous Macrobeam Synthesis
Source: Molecules. 2018 Jul 12;23(7):1701. doi: 10.3390/molecules23071701 (PMC6099500; doi:10.3390/molecules23071701)
Supplement: Supplementary file 1 [file molecules-23-01701-s001.pdf]

Salt-Mediated Au-Cu Nanofoam and Au-Cu-Pd Porous Macrobeam Synthesis

Supporting Information

F. John Burpo[1★]
Enoch A. Nagelli[1]
Lauren A. Morris[2]
Kamil Woronowicz[1]
Alexander N. Mitropoulos[1,3]

[1] Department of Chemistry and Life Science, United States Military Academy, West Point, NY 10996, U.S.A.
[2] Armament Research, Development and Engineering Center, U.S. Army RDECOM-ARDEC, Picatinny Arsenal, New Jersey 07806, U.S.A.
[3] Department of Mathematical Sciences, United States Military Academy, West Point, NY 10996, U.S.A.

★email: john.burpo@usma.edu

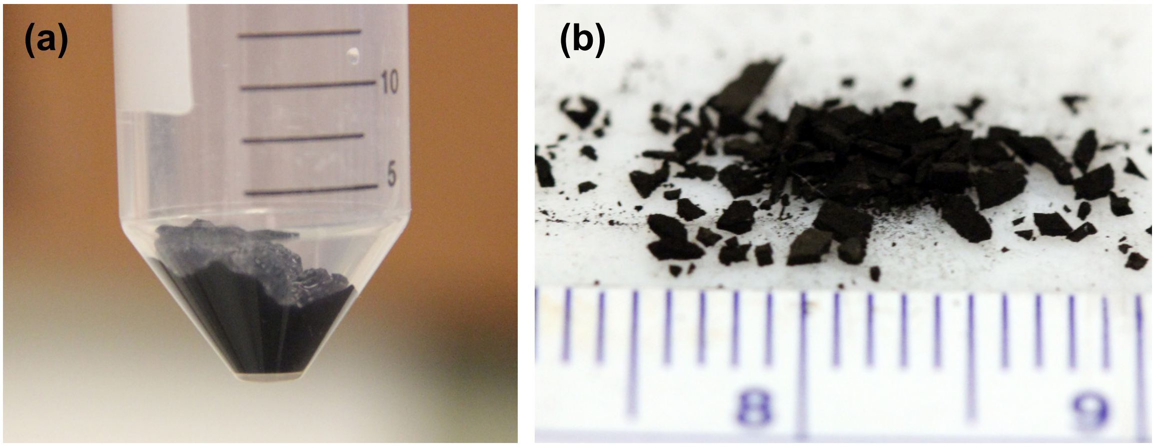

Figure S1. Photograph of Au-Cu nanofoam **(a)** compacted in deionized water, and (b) dried at ambient temperature in air.

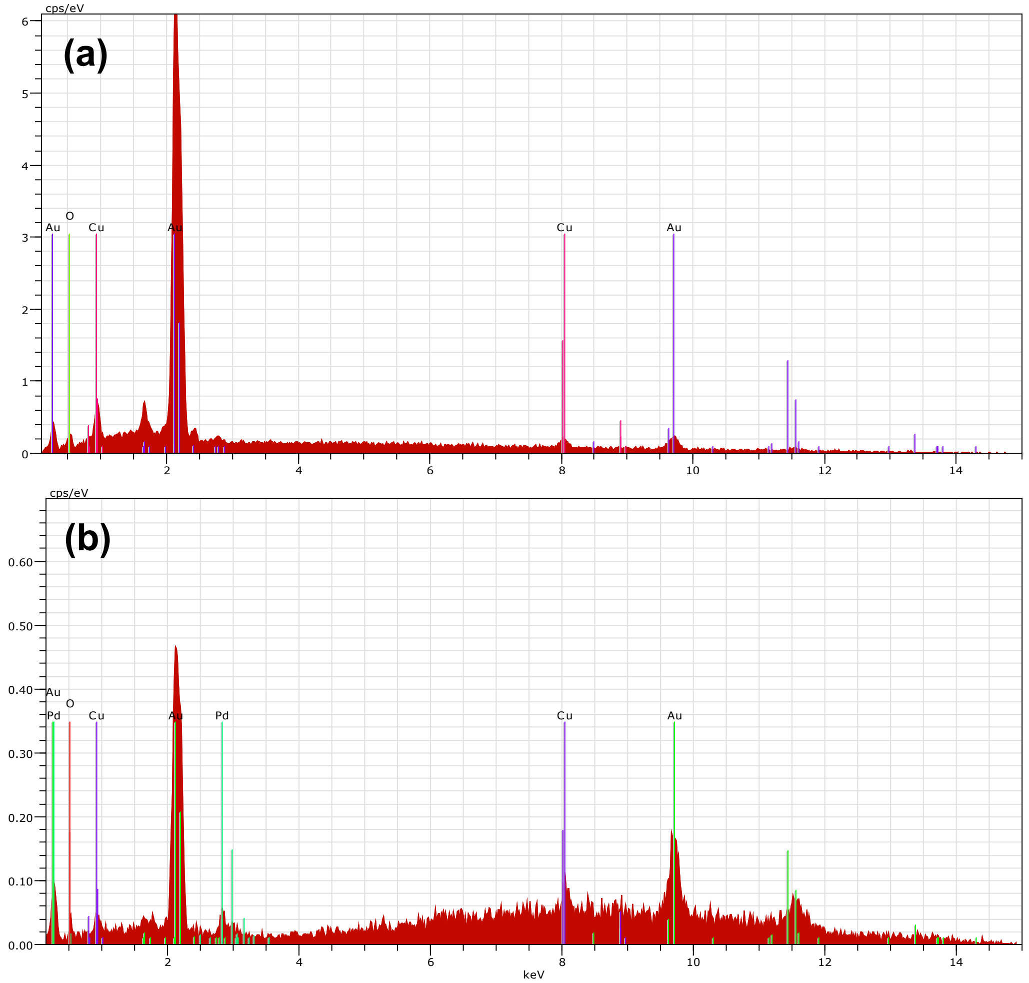

Figure S2. Energy dispersive X-ray spectra (EDS) of **(a)** Au-Cu nanofoams, and **(b)** Au-Cu-Pd macrobeams.

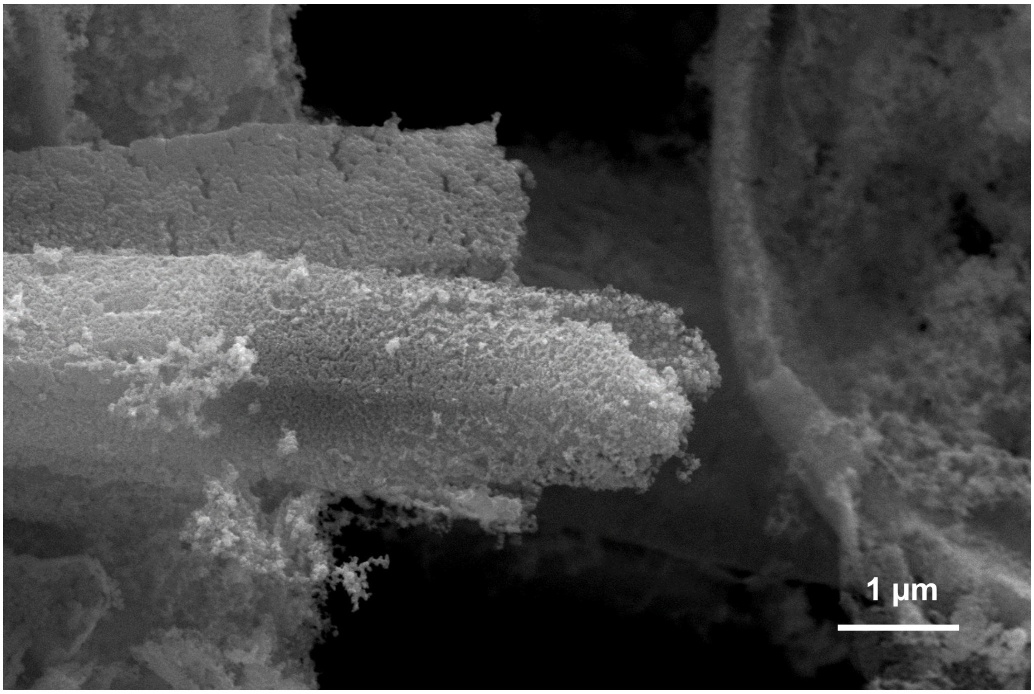

Figure S3. Scanning electron micrograph of Au-Cu-Pd macrobeams with porous sidewalls.

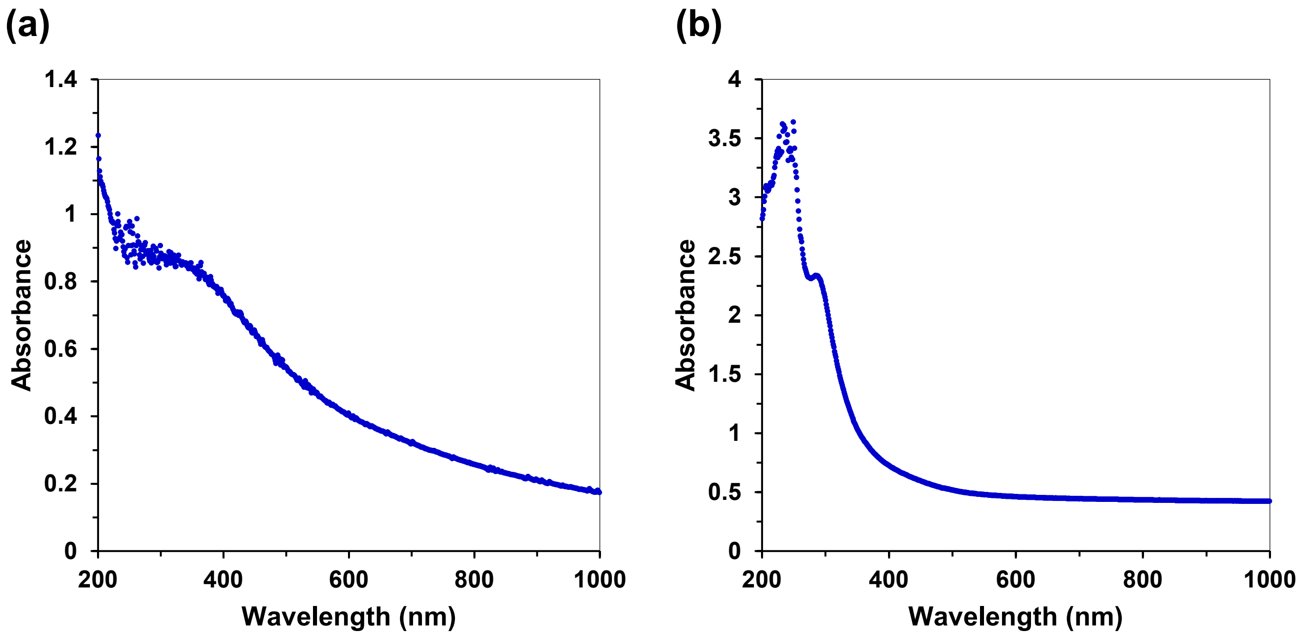

Figure S4. UV-VIS spectra for **(a)** Au-Cu precursor salts, and **(b)** Au-Cu-Pd precursor salts.

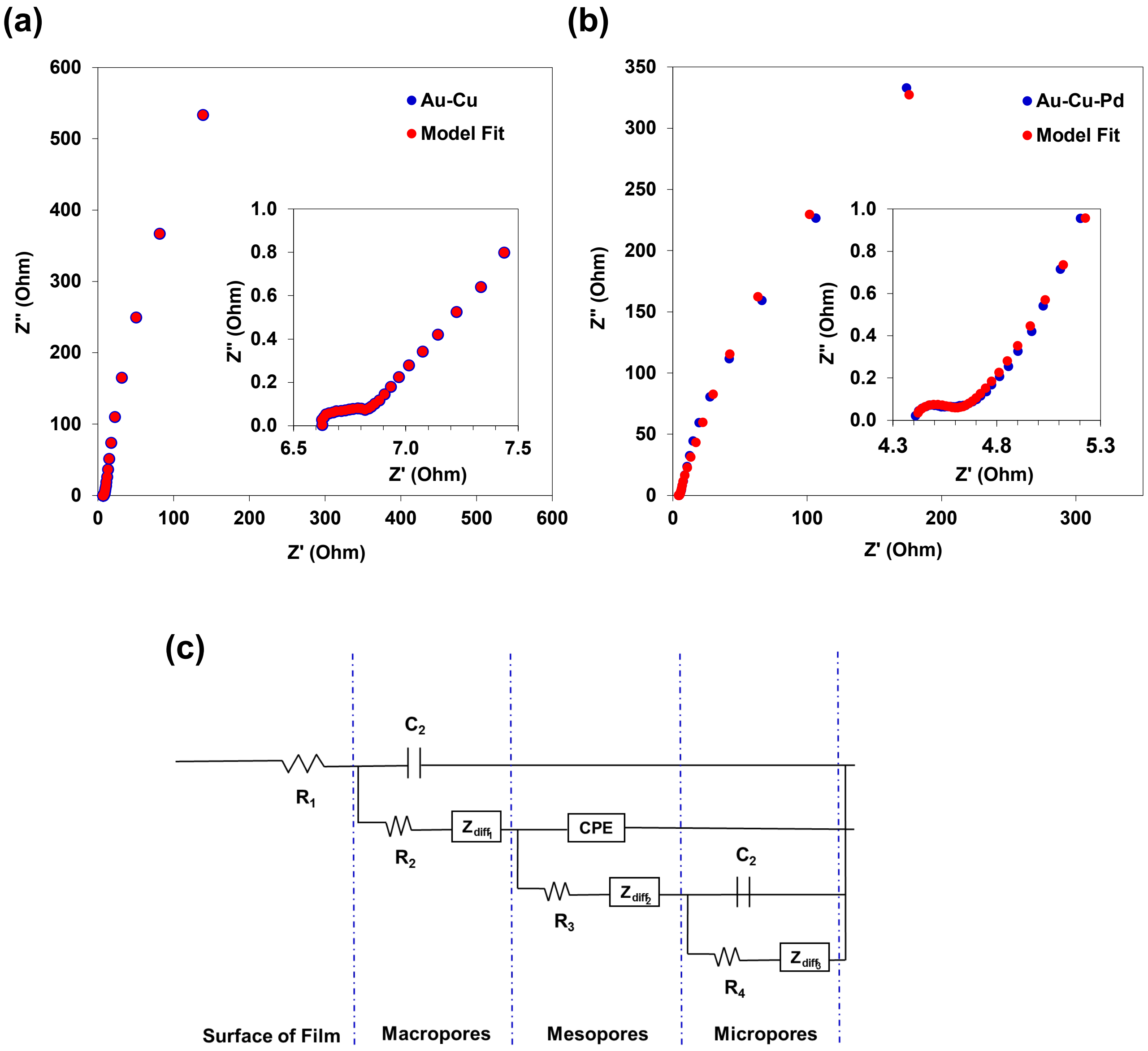

Figure S5. Electrochemical impedance spectra (EIS) and transmission line model (TLM) fitting for **(a)** Au-Cu nanofoams, and **(b)** Au-Cu-Pd macrobeams. **(c)** TLM equivalent circuit model.
